# Inclusion of Real-Time Hand Hygiene Observation and Feedback in a Multimodal Hand Hygiene Improvement Strategy in Low-Resource Settings

**DOI:** 10.1001/jamanetworkopen.2019.9118

**Published:** 2019-08-14

**Authors:** Annick Lenglet, Babette van Deursen, Rebecca Viana, Nura Abubakar, Sarah Hoare, Adebowala Murtala, Mulikat Okanlawon, Jacob Osatogbe, Vera Emeh, Nell Gray, Sara Keller, Pete Masters, Duco Roolvink, Jane Davies, Kaci Hickox, Adolphe Fotso, Karla Bil, Chijioke Ikenna Nwankwo, Bello Ahmad, An Caluwaerts, Isabelle Lessard, Sandrine Dimeglio, Nada Malou, Rupa Kanapathipillai, Melissa McRae, Sidney Wong, Joost Hopman

**Affiliations:** 1 Médecins Sans Frontières, Amsterdam, the Netherlands; 2 Radboud University Medical Centre, Nijmegen, the Netherlands; 3 Médecins Sans Frontières, Abuja, Nigeria; 4 Médecins Sans Frontières, London, United Kingdom; 5 Anka General Hospital, Anka, Nigeria; 6 Noma Children’s Hospital, Sokoto, Nigeria; 7 Médecins Sans Frontières, Brussels, Belgium; 8 Médecins Sans Frontières, Geneva, Switzerland; 9 Médecins Sans Frontières, Barcelona, Spain; 10 Médecins Sans Frontières, Paris, France

## Abstract

**Importance:**

Hand hygiene adherence monitoring and feedback can reduce health care–acquired infections in hospitals. Few low-cost hand hygiene adherence monitoring tools exist in low-resource settings.

**Objective:**

To pilot an open-source application for mobile devices and an interactive analytical dashboard for the collection and visualization of health care workers’ hand hygiene adherence data.

**Design, Setting, and Participants:**

This prospective multicenter quality improvement study evaluated preintervention and postintervention adherence with the *5 Moments for Hand Hygiene*, as suggested by the World Health Organization, among health care workers from April 23 to May 25, 2018. A novel data collection form, the Hand Hygiene Observation Tool, was developed in open-source software and used to measure adherence with hand hygiene guidelines among health care workers in the inpatient therapeutic feeding center and pediatric ward of Anka General Hospital, Anka, Nigeria, and the postoperative ward of Noma Children’s Hospital, Sokoto, Nigeria. Qualitative data were analyzed throughout data collection and used for immediate feedback to staff. A more formal analysis of the data was conducted during October 2018.

**Exposures:**

Multimodal hand hygiene improvement strategy with increased availability and accessibility of alcohol-based hand sanitizer, staff training and education, and evaluation and feedback in near real-time.

**Main Outcomes and Measures:**

Hand hygiene adherence before and after the intervention in 3 hospital wards, stratified by health care worker role, ward, and moment of hand hygiene.

**Results:**

A total of 686 preintervention adherence observations and 673 postintervention adherence observations were conducted. After the intervention, overall hand hygiene adherence increased from 32.4% to 57.4%. Adherence increased in both wards in Anka General Hospital (inpatient therapeutic feeding center, 24.3% [54 of 222 moments] to 63.7% [163 of 256 moments]; *P* < .001; pediatric ward, 50.9% [132 of 259 moments] to 68.8% [135 of 196 moments]; *P* < .001). Adherence among nurses in Anka General Hospital also increased in both wards (inpatient therapeutic feeding center, 17.7% [28 of 158 moments] to 71.2% [79 of 111 moments]; *P* < .001; pediatric ward, 45.9% [68 of 148 moments] to 68.4% [78 of 114 moments]; *P* < .001). In Noma Children’s Hospital, the overall adherence increased from 17.6% (36 of 205 moments) to 39.8% (88 of 221 moments) (*P* < .001). Adherence among nurses in Noma Children’s Hospital increased from 11.5% (14 of 122 moments) to 61.4% (78 of 126 moments) (*P* < .001). Adherence among Noma Children’s Hospital physicians decreased from 34.2% (13 of 38 moments) to 8.6% (7 of 81 moments). Lowest overall adherence after the intervention occurred before patient contact (53.1% [85 of 160 moments]), before aseptic procedure (58.3% [21 of 36 moments]), and after touching a patient’s surroundings (47.1% [124 of 263 moments]).

**Conclusions and Relevance:**

This study suggests that tools for the collection and rapid visualization of hand hygiene adherence data are feasible in low-resource settings. The novel tool used in this study may contribute to comprehensive infection prevention and control strategies and strengthening of hand hygiene behavior among all health care workers in health care facilities in humanitarian and low-resource settings.

## Key Points

**Question:**

Is the implementation of an open-source monitoring and data visualization tool for hand hygiene adherence among health care workers feasible, and does it improve hand hygiene adherence in low-resource settings?

**Findings:**

In this quality improvement study in 2 hospitals in Nigeria, including 686 preintervention and 673 postintervention observations of moments in which hand hygiene was recommended, overall hand hygiene adherence increased from 32.4% to 57.4%.

**Meaning:**

Inclusion of real-time monitoring and data visualization in a standard multimodal hand hygiene improvement strategy was associated with successful implementation and increased hand hygiene adherence in these low-resource settings.

## Teaser

This quality improvement study uses a novel data collection and visualization tool to audit hand hygiene adherence before and after training among health care workers in low-resource settings at 2 hospitals in Nigeria.

## Introduction

Health care–associated infections (HAIs) are a leading concern for patient safety and are associated with prolonged hospital stays, long-term morbidity, increased resistance levels in pathogenic bacteria, higher costs for hospitals and patients, and higher mortality.^1^ In resource-limited settings, the prevalence of HAIs is estimated to be more than 2-fold the prevalence in Europe (15.5 per 100 patients vs 7.1 per 100 patients).^2^ In these contexts, HAIs are also problematic because, as a high proportion of medical care is provided in inpatient departments, antimicrobial-resistant pathogens are highly prevalent, and many patients are particularly susceptible to HAIs (especially neonates and patients who are malnourished, have numerous complex comorbidities, or require surgical care).^3^

Acquisition of an HAI occurs primarily through contact with contaminated hands of transiently colonized health care workers (HCWs) or from contaminated environmental surfaces.^4^ Therefore, hand hygiene for HCWs is the primary method to reduce the spread of HAIs. Unfortunately, hand hygiene adherence rates in HCWs remain low globally.^5,6,7^ To improve hand hygiene adherence, the World Health Organization (WHO) recommends implementing a multimodal approach to strengthening hand hygiene in health care.^8^ This improvement strategy focuses on increasing awareness and adherence of HCWs following the *5 Moments for Hand Hygiene*,^8^ which has proven successful in reducing HAIs in numerous countries.^9,10,11,12^ However, in low-resource contexts (including humanitarian hospitals), there are implementation challenges to increasing HCWs’ adherence. Most hospitals in these contexts lack the appropriate infrastructure and resources to facilitate hand hygiene (eg, insufficient space between beds, crowding of patients), and staff and family caretakers are not trained in appropriate hand hygiene measures and sometimes lack knowledge of the consequences of poor adherence.^3,13^

In humanitarian hospitals, routine audits to measure hand hygiene adherence are infrequent.^3^ Thus, in the absence of simple monitoring tools for hand hygiene, the long-term sustainability and feasibility of hand hygiene strengthening measures are at risk. In higher-resource settings, the current criterion standard for measuring hand hygiene adherence is direct observations of HCWs and performance feedback.^8,14^

Médecins Sans Frontières (MSF) is a medical humanitarian organization that works in diverse health care settings, including outpatient clinics and hospitals. Médecins Sans Frontières’ current infection prevention and control policy focuses, standardizes, and simplifies high-impact infection prevention and control interventions (ie, hand hygiene, cleaning and disinfection, and transmission-based precautions) so that all HCWs are able to understand and prioritize these interventions.^15,16^ For hand hygiene, this includes infrastructure adjustments (eg, more space between beds, availability of clean water and alcohol-based hand sanitizer [ABHS]), training and education for all HCWs, establishing evaluation and feedback mechanisms for adherence, and posters and staff meeting reminders about the importance of the *5 Moments for Hand Hygiene*. As part of improving the evaluation and feedback for hand hygiene adherence, we developed and piloted the use of open-source software on mobile devices and interactive analytical dashboards for the collection and visualization of hand hygiene adherence data in 2 MSF-supported hospitals in northwest Nigeria after hand hygiene strengthening activities. We describe our experiences with the multimodal approach and with the adherence monitoring tools.

## Methods

### Context

The pilot study was conducted from April 23 to May 25, 2018 in 2 hospitals in northwest Nigeria supported by MSF: (1) Anka General Hospital (AGH), Anka, Nigeria, where MSF works with the Nigerian Federal Ministry of Health in the pediatric ward, the inpatient therapeutic feeding center, and the isolation ward, and (2) Noma Children’s Hospital (NCH), Sokoto, Nigeria, where MSF and the Ministry of Health work in preoperative and postoperative wards that care for patients with noma.^17^ The HCWs employed at these sites are a mix of MSF and Ministry of Health employees (ie, nurses, physicians, guards, hygienists, and health promoters). Qualitative data were analyzed throughout data collection and used for immediate feedback to staff. A more formal analysis of the data was conducted during October 2018.

### Hand Hygiene Strengthening Intervention

We implemented infrastructure improvements, educational discussions with HCWs, and a training of trainers (TOT) with supervisory staff at both hospitals.^18^ The infrastructure improvements focused on increasing the availability and accessibility of ABHS at most patients’ beds and in clinical areas. The educational discussions aimed to train HCWs on the rationale, importance, and correct technique of hand hygiene and to understand their existing knowledge, attitudes, and practices about it. The educational discussions were facilitated with different HCW groups using a topic guide that was designed by the study team and included information from previously published hand hygiene topic guides.^19,20^ It covered the following aspects: knowledge about the *5 Moments for Hand Hygiene*, perception of importance and adherence, barriers (including infrastructure), and perception and need for feedback of adherence measurements (eAppendix in the Supplement).

The TOT was conducted as an interactive workshop and covered (1) the MSF infection prevention and control policy, (2) the importance of hand hygiene to prevent HAIs and transmission of multi-drug-resistant bacteria, (3) descriptions and practice of the multimodal strategy and correct hand hygiene techniques, and (4) how to conduct adherence monitoring and feedback. Participants in the TOT were a combination of health promoters, nursing supervisors, nursing activity managers, and water and sanitation supervisors from the hospitals. After the TOT, participants were tasked to provide cascade training to HCWs in each of the wards they were responsible for at the start of their shifts.

### Study of the Intervention

We conducted a prospective multicenter quality improvement study that evaluated an intervention for improving hand hygiene adherence among HCWs. We piloted the use of novel electronic data collection, analysis, and visualization tools for feasibility and ease of use in this lower-resource context. Infrastructure improvements and some training for hand hygiene were performed prior to implementation of this pilot study. For the study, we performed preintervention adherence measurement first, then educational group discussions second, TOT for supervisory staff third, and postintervention adherence measurement last. Discussion participants were sampled purposively, and all participants provided their verbal informed consent to participate in the discussion and for the discussion to be recorded and disseminated. Discussions were transcribed in English by a native English speaker. No data collection was performed during the TOTs. This pilot study fulfilled the criteria for exemption set by the MSF Ethical Review Board for routinely collected data for monitoring and evaluation purposes. This study is reported following the Standards for Quality Improvement Reporting Excellence (SQUIRE) reporting guideline.

### Measures

#### Qualitative Measures

The educational discussions were conducted in English by 1 of us (B.v.D.) with the support of a Hausa-language translator. Groups were composed of HCWs with similar roles, and each discussion lasted 1 hour. In AGH, we conducted 3 educational discussions: 1 with health promoters, 1 with members of the infection prevention and control committee (including nurse ward supervisors, nurses, physicians, nutrition assistants, and laboratory technicians), and 1 with guards and hygiene officers. In NCH, we also conducted 3 educational discussions: 1 with nurses, 1 with ward assistants, and 1 with physicians.

#### Quantitative Measures

**Figure 1.**
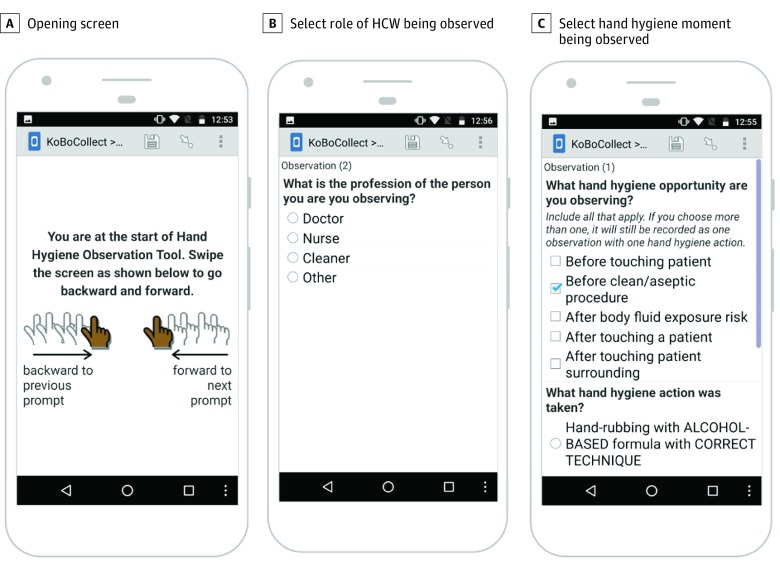
The Hand Hygiene Observation Tool Application and Examples of Different Data Entry Screens on a Mobile Device

Hand hygiene observation data were collected using KoBoCollect (KoBoToolbox), an open-source and free mobile data collection application.^21^ We designed a form in KoBoCollect called the Hand Hygiene Observation Tool (HHOT) (Figure 1). The form was uploaded onto mobile phones with Android operating systems (Alphabet) in AGH and tablets with Android operating systems in NCH. Each observer had participated in the TOT and was trained for approximately 10 minutes on how to use the tool, followed by a supervised observation period. Trained observers recorded data for each directly observed hand hygiene opportunity of HCW staff in the facility. A hand hygiene opportunity was defined as any of the 5 moments defined in the *5 Moments for Hand Hygiene*: (1) before patient contact, (2) before an aseptic or clean task, (3) after bodily fluid exposure risk, (4) after patient contact, and (5) after contact with patient surroundings.^22^ At each opportunity, the observer recorded in the HHOT the number of opportunities for hand hygiene observed for each participant, the HCW role (eg, physician, nurse), and which hand hygiene action they performed. Actions recorded included handwashing with water and soap, hand disinfection with ABHS, wearing gloves, or no action performed. A correct technique was considered as one in which handwashing or ABHS were performed according to WHO protocol. Wearing gloves without either the use of ABHS or handwashing prior to donning gloves was considered incorrect. We defined an adherent hand hygiene opportunity as one that had been conducted correctly. Completed forms were uploaded to a centralized MSF-managed KoBoToolbox server each time the phone or tablet connected to a Wi-Fi signal. The HHOT was used in both preintervention and postintervention measurements.

We conducted preintervention and postintervention hand hygiene adherence direct observations. At least 200 opportunities (per ward, hospital, or role) are needed to obtain reliable results on hand hygiene adherence.^22^ Thus, 200 hand hygiene opportunities were observed in each hospital before the intervention and again after the intervention.^23^ Observations in AGH were conducted in the inpatient therapeutic feeding center and pediatric ward, whereas observations in NCH were conducted in the postoperative ward only. To reduce the burden on the hospitals during the piloting period, we did not conduct the pilot study in all hospital wards. All preintervention and postintervention adherence observations were conducted by 1 of us (B.v.D.), except for the postintervention observations in NCH and the postintervention observations in the pediatric ward in AGH, where 2 other trained observers (S.H. and a nonauthor) conducted the measurement.

In AGH, we conducted the preintervention observations, the implementation of the intervention, and the postintervention measurements within the same week. However, in NCH, we conducted the preintervention measurements and the implementation of the intervention within the same week, and the postintervention measurements were completed within 2 weeks after the TOT.

### Statistical Analysis

#### Qualitative Measures

A simple deductive approach was used to analyze the content of educational discussions (performed by A.L., N.G., and S.K.). This involved reading and rereading the transcriptions and identifying common themes and patterns that emerged in line with the themes highlighted in the topic guide. We also allowed new themes to emerge from the data and cross-checked findings among 3 of us (A.L., N.G., and S.K.) to enhance validity.

#### Quantitative Measures

Hand hygiene adherence was defined as the number of correctly performed hand hygiene actions divided by the number of total hand hygiene opportunities observed.^22^ We measured the adherence at the ward and hospital level and stratified by role level within each hospital.

Using the KoboCollect application programming interface, a set of clearly defined methods for allowing the communication of data among different applications and platforms, we connected data from the KoboToolbox server to Power BI (Microsoft Corp), a partially proprietary online platform for automated data analysis with an interactive visualization display (dashboard). The application programming interface was used to populate Power BI, and the dashboard was refreshed daily.

**Figure 2.**
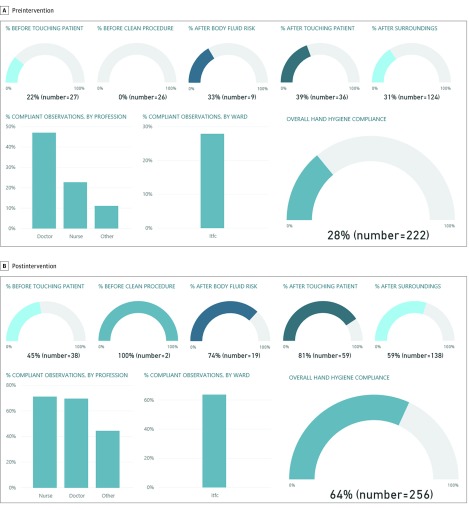
Example of the Hand Hygiene Observation Tool Visualization on an Electronic Interface During the Pilot Study in the Inpatient Therapeutic Feeding Center of Anka General Hospital, Anka, Nigeria

Figure 2 shows an example of the dashboard representation of one of the adherence measurements conducted in AGH. The dashboard was designed to calculate adherence by time period, facility, ward, HCW profile, or any of the 5 moments. Through interactive clicking, visualization of the observations are stratified by HCW (eg, physicians, nurses), ward, and time period when the observations were performed (day, month, and year). The absolute number of observations are presented with respective proportional calculations of the total number of observations (Figure 2).The target audience for the dashboard included the HCWs and management and supervisory staff at both hospitals.

To evaluate whether the intervention was associated with hand hygiene adherence, we compared preintervention with postintervention adherence rates and calculated 2-tailed χ^2^ and their respective *P* values to determine significance of the difference. Statistical significance was set at less than .05. These analyses were conducted using Stata statistical software version 15 (StataCorp).

## Results

Health care workers felt that the importance of hand hygiene was associated with preventing transmission of infections to the individual (Table 1). Understanding of the 5 moments was not necessarily the same as those outlined by WHO in *5 Moments for Hand Hygiene*. Participants listed several other important moments: before entering the hospital, after going to the toilet, and before praying. Most medical HCWs (ie, nurses and physicians) reported that they should perform hand hygiene before touching a patient, after bodily fluid exposure, and after touching a patient. However, the moments before an aseptic procedure and after touching a patient’s surroundings were not frequently mentioned. Gloves were mentioned often as a method of hand hygiene, and there was confusion around when one should use gloves compared with washing one’s hands or using ABHS.

**Table 1.** Representative Quotations From Health Care Workers in Educational Discussions Regarding Hand Hygiene in AGH and NCH

Topic	Quotation	Source (Hospital)
Importance of hand hygiene	“…we do everything with the hand. That is the reason why it is important for hand hygiene”	Guard (AGH)
	“…to prevent infection transfer from patient to patient or from patient to yourself or from patient to outside the hospital or the community.”	Physician (NCH)
	“…for me, it is important to do hand hygiene. In order to stop the transmission, you should wash your hands. And there are a lot of patients with diarrhea and you can spread it with your hands through the mouth and fecal route. For example, cholera.”	Health promoter (AGH)
WHO *5 Moments for Hand Hygiene*	“I think people don’t know the 5 moments....”	Health promoter (AGH)
Gloves as a method of hand hygiene	“The gloves that we use are single used. So one pair for the patient, and one pair for [the next] patient.”	Health promoter (AGH)
Barriers to hand hygiene	“Too many patients with just one nurse.”	Nurse (NCH)
	“Not only for the staff. Also for the patients themselves.”	Physician (NCH)
Alcohol-based hand sanitizer	“Not around all the time. Sometimes they [alcohol-based hand sanitizer supply] got finished before it will be replaced.”	Physician (NCH)
	“But for me, sometimes I use the alcohol swab and the hand rub. I feel comfortable, I still need still to wash my hand, like, even if I want to eat something, I still feel like there is something there.”	IPC committee member (AGH)
Reminders for hand hygiene	“…second thing is that you know, as human beings, people tends to [inaudible] at times, so we need a reminder.”	Physician (NCH)

Abbreviations: AGH, Anka General Hospital; IPC, infection prevention and control; NCH, Noma Children’s Hospital; WHO, World Health Organization.

The barriers to performing hand hygiene highlighted by HCWs were ABHS being unavailable or not easily accessible in clinical areas, absence of both water and soap at handwashing stations, and large patient loads and thus lack of time to perform hand hygiene between patients (Table 1). Several participants mentioned that we should include patients and caretakers in hand hygiene training sessions. The ABHS was also mentioned as a deterrent because people felt that they needed to wash their hands after having used it owing to their hands feeling dirty (Table 1). Finally, the need for reminders, posters throughout the hospital, and more training sessions for all staff levels that work in the hospital was mentioned in most groups.

Overall, hand hygiene adherence increased from 32.4% before the intervention to 57.4% after the intervention. Lowest overall postintervention adherence occurred before patient contact (53.1% [85 of 160 moments]), before aseptic procedure (58.3% [21 of 36 moments]), and after touching a patient’s surroundings (47.1% [124 of 263 moments]).

In the AGH inpatient therapeutic feeding center, we conducted 222 preintervention hand hygiene adherence observations and 256 postintervention observations; the overall adherence was 24.3% (54 moments) before the intervention and 63.7% (163 moments) after the intervention (*P* < .001). Before the intervention, physicians had a higher adherence rate (47.2% [25 of 53 moments]) than nurses (17.7% [28 of 158 moments]). However, after the intervention, the adherence rates for both physicians and nurses were similar (physicians, 69.9% [55 of 79 moments]; nurses, 71.2% [79 of 111 moments]). The lowest hand hygiene adherence rates after the intervention were for the moments before touching a patient (44.4% [17 of 28 moments]) and after touching a patient’s surroundings (59.4% [82 of 138 moments]) (Table 2).

**Table 2.** Preintervention and Postintervention Hand Hygiene Adherence Stratified by Ward and Hospital

Adherence	No./Total No. (%)					
	NCH Postoperative Ward		AGH ITFC		AGH Pediatric Ward	
	Preintervention	Postintervention	Preintervention	Postintervention	Preintervention	Postintervention
Overall	36/205 (17.6)	88/221 (39.8)^a^	54/222 (24.3)	163/256 (63.7)^a^	132/259 (50.9)	135/196 (68.8)^a^
Role						
Physician	13/38 (34.2)	7/81 (8.6)^a^	25/53 (47.2)	55/79 (69.6)^a^	62/99 (62.6)	52/53 (82.5)^a^
Nurse	14/122 (11.5)	78/126 (61.4)^a^	28/158 (17.7)	79/111 (71.2)^a^	68/148 (45.9)	78/114 (68.4)^a^
Cleaner	NR	NR	0/2	0/1	NR	6/17 (35.3)
Other^b^	9/45 (20.0)	3/13 (23.1)	1/9 (11.1)	29/65 (44.6)	1/8 (12.5)	1/2 (50.0)
Moment						
Before patient contact	1/31 (3.2)	29/74 (39.2)^a^	5/27 (18.5)	17/38 (44.4)^a^	23/47 (48.9)	39/48 (81.3)^a^
Before aseptic procedure	1/16 (6.3)	2/2 (100)^a^	0/26	2/2 (100)^a^	0/4	17/32 (53.1)
After bodily fluid exposure risk	7/32 (21.9)	8/10 (80.0)^a^	1/9 (11.1)	14/19 (73.7)^a^	9/20 (45.0)	13/18 (72.2)
After patient contact	11/33 (33.3)	38/64 (59.4)^a^	13/36 (36.1)	48/59 (81.4)^a^	36/52 (69.2)	36/44 (81.8)
After touching a patient’s surroundings	16/93 (17.2)	11/71 (15.5)	35/124 (28.2)	82/138 (59.4)^a^	63/136 (46.3)	31/54 (57.4)

Abbreviations: AGH, Anka General Hospital; ITFC, inpatient therapeutic feeding center; NCH, Noma Children’s Hospital; NR, none recorded.

^a^ *P* < .05.

^b^ Includes mental health staff, nutrition assistants, and physiotherapists.

In the AGH pediatric ward, we conducted 259 preintervention hand hygiene adherence observations and 196 postintervention observations. Adherence increased from 50.9% (132 of 259 moments) before the intervention to 68.8% (135 of 196 moments) after the intervention (*P* < .001). Physicians had the highest adherence rate before the intervention (62.6% [62 of 99 moments]) and after the intervention (82.5% [52 of 53 moments]) among all roles. Nurses’ adherence rate increased from 45.9% (68 of 148 moments) to 68.4% (78 of 114 moments) between the preintervention and postintervention periods (*P* < .001). The increase in adherence was also seen in each of the 5 moments. After the intervention, the lowest adherence rates were for the moments before aseptic procedures (53.1% [17 of 32 moments]) and after touching a patient’s surroundings (57.4% [31 of 54 moments]) (Table 2).

At NCH, we conducted 205 preintervention hand hygiene observations and 221 postintervention observations. The overall adherence increased from 17.6% (36 moments) to 39.8% (88 moments) between the preintervention and postintervention periods (*P* < .001). Before the intervention, physicians had a higher rate of hand hygiene adherence than nurses (34.2% [13 of 38 moments] vs 11.5% [14 of 122 moments]). However, after the intervention, the adherence rate among physicians decreased to 8.6% (7 of 81 moments) (*P* < .001), whereas that of nurses increased to 61.4% (78 of 122 moments) (*P* < .001). Adherence at each of the 5 moments increased between the preintervention and postintervention periods. However, adherence remained less than 50% for the moments before patient contact (39.2% [29 of 74 moments]) and after touching a patient’s surroundings (15.5% [11 of 71 moments]) (Table 2).

## Discussion

Our results suggest that the implemented multimodal hand hygiene improvement strategy can be successful in increasing HCW hand hygiene adherence, as demonstrated in these 2 hospitals in Nigeria. Additionally, we were able to successfully develop and use an open-source mobile data collection tool to perform hand hygiene observations and create an automated dashboard to support data display and analysis as well as feedback to HCW staff. These tools are useful for further extrapolation in different MSF settings but can also be easily replicated by other organizations in low-resource settings. Also, our study contributes to the lack of research around hand hygiene adherence in these contexts.^24^

We observed that hand hygiene adherence differed between nurses and physicians. Particularly, the decrease in postintervention adherence in the physician group at NCH was concerning. This specific group of HCWs may benefit from a more targeted training on hand hygiene. We also observed that the *5 Moments for Hand Hygiene* (particularly after touching a patient’s surroundings) were inconsistently understood by the groups of HCWs. These differences in knowledge and adherence with regard to hand hygiene among different HCWs are well documented.^25,26,27^ The differences in adherence observed between AGH and NCH might have been owing to AGH already having initiated some preliminary hand hygiene improvement activities before the pilot study, including placing posters and reminders about the 5 moments in the wards and increasing the availability and access to ABHS. In NCH, only some of these preliminary hand hygiene improvement activities had commenced at the time of the pilot study; thus, the staff were less sensitized. Health care workers in AGH and NCH expressed the importance of increased access to ABHS, presence of functioning handwashing stations, and reminders and ongoing training on hand hygiene for staff, patients, and caretakers. These aspects have all been shown to have a positive effect on hand hygiene adherence among HCWs.^25,26,27,28^

The uses of technological approaches to improve hand hygiene adherence in health care settings have been well documented in mostly higher-resource settings.^29^ Recent studies have shown that using electronic badges that trigger personalized hand hygiene messages for HCWs^30^ or badges that record entry and exit into specific areas and whether the individual used an ABHS dispenser^31^ are effective for improving and sustaining hand hygiene adherence in health care settings. However, a 2015 systematic search^32^ for mobile applications that support the prevention of HAIs (eg, guidelines, monitoring for hand hygiene) concluded that there was a severe shortage of such tools.

This was a pilot evaluation of the HHOT. However, the findings have direct applications to routine activities focusing on improving hand hygiene in health care settings. The HHOT is free and can easily be adopted (and adapted) to any health care setting to monitor hand hygiene adherence. The HHOT allows for more discrete and faster adherence observations, decreases use of paper, eliminates the need for data entry, and could improve data quality. Although the HHOT should only be used by a trained observer, it is easy to use and teach and works well for adherence monitoring in low-resource settings. Also, the dashboard allows quick analysis of data by ward, HCW role, and the 5 moments, thus facilitating the rapid identification of low adherence. This information can be used to rapidly target interventions and educational efforts for HCW groups or specific hand hygiene moments. The version of PowerBI used by MSF is a paid version, but a simplified free version also exists. Even so, data collected in the HHOT can easily be shared with any free or open-source data analytics system or program to generate automated, real-time, data visualization dashboards (eg, MSF’s Github dashboard [https://github.com/MSF-UK/MSF-Dashboard]). The only costs associated with customizing an electronic dashboard would be from development work.

### Limitations

Our intervention and pilot study had some limitations. The educational discussions were useful in starting a dialogue about hand hygiene with staff in the 2 hospitals; however, researcher time was limited, and the duration of the sessions was too short to serve as a qualitative investigation regarding perceptions and barriers to hand hygiene and as a teaching opportunity to increase awareness and adherence. However, the topic guide served as a useful tool for future interventions and as a starting point for future dialogues with HCWs. It is currently being further developed for use as a teaching tool (rather than a research tool) for health care facilities to address hand hygiene in an interactive manner. This was a pilot study with a quasi-experimental design (ie, without a control group) that was conducted within a short time. As such, the results cannot be extrapolated to other settings and may not be representative of possible improvements in hand hygiene adherence in AGH or NCH for the longer term. After the pilot of the HHOT, monthly adherence measurements have continued as part of the multimodal strengthening initiatives in both hospitals. We are aware that without ongoing efforts and investments in hand hygiene, it is likely that adherence rates will decrease through time in both hospitals. The HHOT relies on direct observations of hand hygiene adherence, which, despite being the criterion standard,^14,22^ is inherently subject to the Hawthorne effect.^33^ We tried to limit this bias by using a mobile device for observations (which could suggest that the observer was looking at his or her smartphone rather than observing the participant). In both hospitals, preintervention and postintervention observations were conducted by 2 different observers, which may have altered the way in which adherence was judged. This was minimized by ensuring the second observer was well trained and supervised by another trained individual. Additionally, the HHOT relies on a Wi-Fi internet connection to upload data to the server. This might not be appropriate in all low-resource hospitals where the internet is not always available. However, there are technological solutions to counter this challenge for these settings, such as installing local servers.

## Conclusions

Improving hand hygiene in health care settings requires a multimodal approach, including education, observations, supplies, feedback about adherence to staff, and directives on implementing hand hygiene.^34^ The MSF hand hygiene improvement strategy incorporates all of these aspects. The use of an application-based adherence monitoring tool with a real-time dashboard could contribute further to this strategy and enhance HCW engagement and buy-in in hand hygiene strengthening activities in their health care facilities. Furthermore, it improves standardization and sustainability of hand hygiene improvement strategies, both of which are crucial in settings with low resources.

## Notes

Supplement. **eAppendix.** Methods
