# Supplementary material for: Inclusion of Real-Time Hand Hygiene Observation and Feedback in a Multimodal Hand Hygiene Improvement Strategy in Low-Resource Settings
Source: JAMA Netw Open. 2019 Aug 14;2(8):e199118. doi: 10.1001/jamanetworkopen.2019.9118 (PMC6694391; doi:10.1001/jamanetworkopen.2019.9118)
Supplement: Supplement. — eAppendix. Methods [file jamanetwopen-2-e199118-s001.pdf]

Supplementary Online Content

Lenglet A, van Deursen B, Viana R, et al. Inclusion of real-time hand hygiene observation and feedback in a multimodal hand hygiene improvement strategy in low-resource settings. *JAMA Netw Open*. 2019;2(8):e199118. doi:10.1001/jamanetworkopen.2019.9118

eAppendix. Methods

This supplementary material has been provided by the authors to give readers additional information about their work.

© 2019 Lenglet A et al. *JAMA Network Open.*

eAppendix. Methods

TOPIC GUIDE FOR EDUCATIONAL GROUP DISCUSSIONS

<u>Please note:</u>

- This guide is to help guide the topics to be explored during this group discussion;
- It is not an exhaustive list of questions. The order and wording of questions will be adapted based on the topics and priorities identified by the participant, also aiming to ensure a natural and informal flow to the conversation.
- Each group's discussion is anticipated to last up to one hour.
- The following introduction and conclusion will be used for each of the following topic guides.

Introducing the group discussion

- Thanking participants for considering taking part in the study.
- Introduce yourself and what you do
- Tell the participants:

We would like to talk to you about hand hygiene practices here in Anka pediatric ward/Noma Children's hospital. When we say hand hygiene, we mean everything associated with either disinfection or cleaning of your hands during your working day. We would like to hear your stories, what you think is working well and the challenges you face. We hope that by gathering opinions and ideas here, you will have a better understanding of what hand hygiene is, but also that we can better understand the challenges you face in your daily work to adhere to the 'guidelines' around this. The discussion will take between 45 minutes to one hour. I will be recording the group discussion so I can make sure not to miss anything that was said. If at any point you would like to take a break or stop, just let me know. If you would like to continue there is a form we have to complete to check that you have all the information you need before we start. Would you like to continue?

- If participant(s) agrees, go through information sheet and consent process (see information sheet and consent form).
- Emphasize that this discussion is confidential: nothing that is discussed will be repeated outside the group and we ask all participants to also respect this confidentiality and not discuss comments made outside the group.
- Ask the participants not to mention names of others during the discussion; if you wish to refer to the actions of another you can but as 'a staff member…' or similar.
- Emphasize that it is not a test: there are no right or wrong answers – we only want to understand the reality in order to best improve hand hygiene. Whatever you say here will have no implications (positive or negative) for you or your work with MSF.
- Ask the participant(s) if s/he/they has any questions.
- Ask the participant(s) if s/he/they is/are comfortable to begin.
- Confirm start of audio recording.

© 2019 Lenglet A et al. *JAMA Network Open.*

Topic guide

Question	Prompt	Notes
**How do you define hand hygiene?**<br>**(**True or false: post-its with correct and incorrect stamens of HH are distributed. Participants are asked place it on flip-chart on true/false part of sheet)	• Focus on true and false activity	• Remember to take picture of flipchart before explanation

© 2019 Lenglet A et al. *JAMA Network Open.*

Knowledge about hand hygiene and the 5 moments

**Question**	**Prompt**	**Notes**
**Why do you think it is important to perform hand hygiene?** (~ 10min)	Anything else?	
**At work/hospital, when do you think you should be performing a 'hand hygiene activity'** (20 min)	• At which moments do you use alcohol gel?<br>• At which moments do you wash your hands?<br>• At which moments do you wear gloves?<br>• Explain when you should use alcohol gel and when you should do water and soap. Change according to the 5 moments of hand hygiene	• Detergent; alcohol gel and gloves displaced at desk to facilitate discussion.
**Where do you think you should perform hand hygiene?** (5 min) (Show picture of hospital room without the arrows – and ask people to point at where/when they would do hand hygiene in this setting)	• Explain each of the 5 moments of hand hygiene and show the correct diagram	• Spend more time with explanation; ask if they have any questions
**What is needed to perform correct hand hygiene?**	• Should you dress specifically?	

Compliance and perception of hand hygiene

**Question**	**Prompt**	**Notes**
**Were you aware of the 5 moments of HH?**<br>**What challenges do staff here face complying with hand hygiene guidelines?**<br>**Do you think you are adhering to the guidelines for hand hygiene?**<br>**If I told you that studies shows that medical doctors have the lowest compliance to HH would you be surprised?**	• Are you compliant with each of the 5 moments?<br>• If yes, why?<br>• If no, why not?<br>• Which ones are less easy to be compliant to and why?<br>• What motivates you to be compliant and why?	• If necessary, make it indirect question: are the "5 moment for HH" part of your routine with every patients?

© 2019 Lenglet A et al. *JAMA Network Open.*

Barriers around hand hygiene (including structural considerations)

**Question**	**Prompt**	**Notes**
**What would improve your compliance with hand hygiene guidelines?**<br>*What would make it easier for you to be compliant and why?*	• Is there enough alcohol rub available?<br>• Is alcohol based handrub available at bedside/patient zone?<br>• Are there sinks with running water and soap available where you need them?<br>• How do you feel about using alcohol rub? How does it make your hands feel?	

© 2019 Lenglet A et al. *JAMA Network Open.*

Observations around HH compliance

Question	Prompt	Notes
**Introduction to hand hygiene compliance**	In the last few days, you will have seen someone walking around the hospital with a phone and entering in data into the phone. They have been observing all the possible moments of hand hygiene that occurred in the ward during this time, whether hand hygiene was performed in those moments and whether it was performed correctly. This will allow us to calculate a percentage of "compliance" to hand hygiene standards. The aim will be to reach 100% compliance. However, we also know that this will be extremely difficult to achieve.	
**How did you feel about "being observed" these last few days?**	• What is your experience?<br>o Was it a pleasant experience?<br>o Was it an unpleasant experience?<br>• Did it interfere with your work?	
**We can now calculate a percentage of compliance. What would be your preferred way of receiving feedback about the compliance number, what went well and what could have been done better?**	• Would you prefer immediate individual feedback (both positive and negative) after an observation?<br>• Would you prefer individual feedback at the end of the day (in private or in front of colleagues?)?<br>• Would you prefer receiving collective feedback (i.e. to all staff?)?<br>• If group feedback, which format would you like such feedback to be presented?<br>• Medical meeting?<br>• Morning staff meeting?<br>• Posters in the ward?<br>• Public report on the door of the hospital?	

Concluding the group discussion

Interviewer summarizes the discussion and highlights the core of the discussion.

- *Of everything people discuss today, what has been most important for you?*
- *Is there anything you would like to add to this discussion, or anything that we missed?*
- *Do you have any questions you would like to ask me?*
- *Thank you for your time!*

© 2019 Lenglet A et al. *JAMA Network Open.*
